# Innovative Carbon Black Replacement in Rubber Compound: Impact of Pyrolytic Carbon Black and Energy-Gypsum By-Products on Vulcanization and Properties

**DOI:** 10.3390/polym17223080

**Published:** 2025-11-20

**Authors:** Ivan Labaj, Juliána Vršková, Ivan Kopal, Andrej Dubec, Darina Ondrušová

**Affiliations:** Faculty of Industrial Technologies in Púchov, Alexander Dubček University of Trenčín, Ivana Krasku 491/30, 020 01 Púchov, Slovakia; juliana.vrskova@tnuni.sk (J.V.); ivan.kopal@tnuni.sk (I.K.); andrej.dubec@tnuni.sk (A.D.); darina.ondrusova@tnuni.sk (D.O.)

**Keywords:** rubber compounds, pyrolytic carbon, alternative filler, energy-gypsum

## Abstract

This study focuses on the possibility of substituting the conventional carbon black filler N339 in a rubber compound, which also contains an energy-gypsum filler obtained as an industrial by-product, with an alternative carbon filler produced by pyrolysis of rubber waste. The proposed basic rubber compound recipe demonstrated excellent mechanical properties, which were also verified through industrial tests. Practical application in transport systems confirmed the reliability, durability, and robustness of the compound ensuring long-term functionality even in demanding operating conditions. From an ecological point of view, the substitution of conventional fillers with pyrolytic carbon materials is being pursued to reduce the environmental burden of the material. The rheological and vulcanization characteristics of the compounds were subject to minimal changes of up to 8%. The increase in the scorch time value of the compounds can be positively assessed given that the optimal vulcanization time has decreased, which leads to a faster course of the vulcanization process. The most significant effect was a 56% reduction in tensile strength without significant effect on elongation at break. These results document the potential for complete replacement of traditional fillers in rubber systems while maintaining several functional parameters.

## 1. Introduction

Rubber compounds as well as fillers have gone through a long development path until now. In the case of rubber compounds, today more attention is paid to their ecological side, carbon footprint, and recycling [1,2,3,4,5,6]. It is precisely these aspects of development that bring to the fore the so-called alternative fillers, which often come from waste from other branches of industry, or by-products without massive application. The field of alternative fillers is becoming one of the most current topics in materials science and elastomer technology in the 21st century, with several scientific publications focusing on their development, surface treatment, and use [7,8]. This interest stems from the need to replace traditional fillers with more ecological and efficient materials that also improve the mechanical and functional properties of rubber compounds. Research into alternative fillers supports innovation in the field of sustainable and high-performance rubber compounds, which has a significant impact on industry and environmental policy. Almost every filler for the rubber industry was an alternative at the beginning of its use, and with the passage of time and widespread use, it became common. Moreover, all industries in today’s world are influenced by the development of new types of materials and the improvement of the properties of their products [9,10,11,12].

Nowadays, the label alternative filler belongs mainly to those that have the above-mentioned origin (waste from other branches of industry, or a by-product), mainly because of their lower carbon footprint. Their footprint is included in the overall carbon footprint of production, so it is not necessary to produce them through special processes, a concept often referred to as closing the production cycle (application of all waste without the need for landfilling or energy recovery) [13,14,15]. The application of alternative fillers in industrially used rubber compounds requires a complex and long-term study of their influence on the mixing process and properties of the vulcanizate, as well as the adjustment of recipes. Most alternative fillers, due to their origin, often have a variable chemical composition (depending on the input materials, the process conditions in which they are formed, or the effect of additional processing), which is one of their main disadvantages compared to commonly used inorganic, conventional, industrially produced fillers. In addition to the chemical composition, a disadvantage of alternative fillers is their particle size, which requires additional processes to achieve a size suitable for use in the rubber industry without significant negative impacts on the properties of rubber compounds and vulcanizates [16,17,18].

One of the widely discussed alternative fillers is pyrolytic carbon black (PCB), which has the potential to replace commonly used carbon black (CB) in the future [19,20]. Many publications deal with the possibility of replacing (completely, partially) commonly used industrial fillers with an alternative carbon-based filler that is generated as waste during the pyrolytic processing of polymer, industrial, and rubber waste. In article [21], the CB content of N660 was replaced with PCB, with the results showing that up to 20% of the CB content of N660 can be replaced, without a negative impact on the mechanical and physical properties of the rubber compound. One of the studies [22] dealing with the replacement of CB N330 with PCB reported that compounds containing PCB showed only a slight decrease in mechanical properties but improved resistance to accelerated aging. Similar conclusions were also described in the article [23] dealing with the replacement of N550 carbon black with PCB, where no significant changes in mechanical properties were observed after heat-aging. The author recommended the use of PCB as a supplementary filler to commercial N550. In another study [24], PCB is compared to N770 carbon blacks, especially with respect to rheometric and mechanical properties. One of the disadvantages is described as the ash content of PCB, which is 20 times higher compared to conventional carbon blacks.

## 2. Materials and Methods

### 2.1. Rubber Compound Preparation

**Figure 1:**
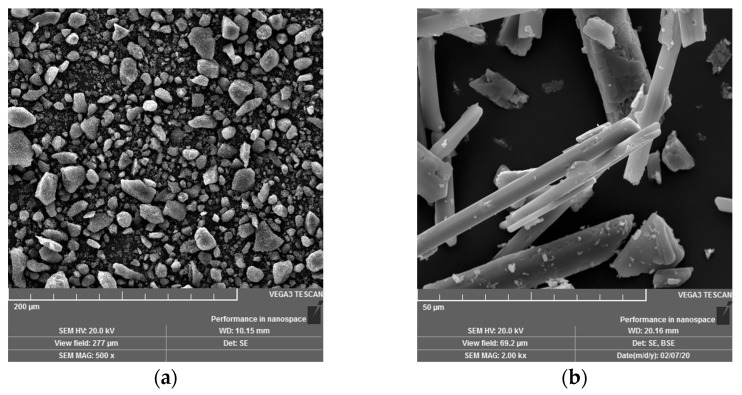
Morphology of alternative fillers used in the study, (**a**) PCB, mag. 500×, (**b**) EG, mag. 2000×.

The rubber compounds were prepared according to the recipe given in Table 1. All modifications of the recipe using pyrolysis carbon black (PCB) were based on the basic recipe (BB) containing the alternative filler energy-gypsum (EG), in which the commonly used filler carbon black N339 (CB) (Makrochem, Lublin, Poland) was gradually replaced by PCB (25, 50, 75, 100%). For all prepared compounds, a PCB filler fraction smaller than 25 μm was used. The chemical composition of the fillers used is given in Table 2, and the morphology of alternative fillers (PCB, EG) is presented in detail using SEM images (Figure 1), which allow visualization of the shape, size, and surface structure of individual particles. Natural rubber type SMR 10 (Lee Rubber, Kuala Lumpur, Malaysia) was used as a matrix for all prepared rubber compounds. Zinc oxide (ZnO) (SlovZink a.s., Košeca, Slovakia) and stearic acid (SA) (Setuza a.s., Ústí nad Labem, Czech Republic) were chosen as sulfur vulcanization activators. The vulcanization system itself consisted of the vulcanization accelerator N-tert-butyl-2-benzothiazole sulfonamide (TBBS) (Duslo a.s., Šaľa, Slovakia) and the vulcanization agent sulfur Crystex OT33 (Eastman Chemical company, Kingsport, TN, USA). 

The mixing process was carried out on a laboratory mixer Brabender Plastograph EC Plus (Brabender GmBH & Co.KG, Duisburg, Germany) with a chamber volume of 80 cm^3^, at set mixing conditions of 90 ± 1 °C and at a rotor blades speed of 50 ± 1 rpm. Using the WinMix program, measurable parameters of the mixing process were recorded simultaneously during the mixing process, namely the torque and the real temperature of the mixed compound, the output of which are curves characterizing the mixing process of individual compounds. After the mixing process, additional homogenization was performed using a laboratory double-cylinder LaboWalz W150 (Voght Labormaschinen GmBH, Berlin, Germany) with subsequent standing of the compound for 24 h at laboratory temperature.

### 2.2. Vulcanization Parameters and Process

For each of the prepared compounds, rheological properties and vulcanization characteristics were determined using RPA 2000 (Alfa Technologies Ltd., Akron, OH, USA). The test parameters were set at 160 °C (vulcanization temperature) and 20 min (test time). The vulcanization of the rubber compounds was carried out on a Fontijne LabEcon 600 vulcanization press machine (Fontijne Presses, Vlaardingen, Netherlands) according to the optimal vulcanization time of each compound in the form of a plate with a thickness of 2 mm for determining the tensile properties (ISO 37) [25] and a ring with a diameter of 50 mm and a thickness of 6 mm for determining the hardness (ISO 48) [26].

For verification of the behavior of individual fillers during the vulcanization process, compounds without a vulcanizing agent (sulfur) were prepared (Table 3), each containing only one filler so that its specific effect could be precisely identified. Test parameters were set identically as in the case of the complete compounds (Table 2).

### 2.3. Payne Effect

The Payne effect was determined using RPA 2000 (Alfa Technologies Ltd., Akron, OH, USA) on unvulcanized compounds at a temperature of 70 °C, a frequency of 1 Hz, and varying strains in the range of 0.28–100%.

### 2.4. Hardness Determination

After 24 h of vulcanization, the hardness (ISO 48) was determined using a digital hand-held hardness tester with a Shore A scale Bareiss HPE II (Bareiss, Stouffville, ON, Canada) [26]. The measurement was performed on samples with a thickness of 6 mm, and the measurement was repeated 6 times on each sample. The measured values were statistically processed.

### 2.5. Tensile Properties

From the prepared plates of rubber compounds, test specimens in the shape of a dumbbell of type A2 (ISO 37) were cut out on an Instron cutter machine (Instron, Norwood, MA, USA) [25]. A universal tensile tester, Shimadzu AG-X Plus (Shimadzu, Tokyo, Japan), was used to determine the tensile properties (tensile strength, ductility). The set tensile test parameters were as follows: gauge length 50 mm and test speed 500 mm/min.

### 2.6. Morphology Analysis

The cross-sectional morphology of the compounds was observed using a scanning electron microscope (SEM, Model: VEGA 3, Tescan, Czech Republic) in the secondary electron mode. A representative area was selected (considering the size of the filler particles) from the large surface, which reliably reflects the morphology of the entire region.

## 3. Results and Discussion

### 3.1. Mixing Process Parameters

**Figure 2:**
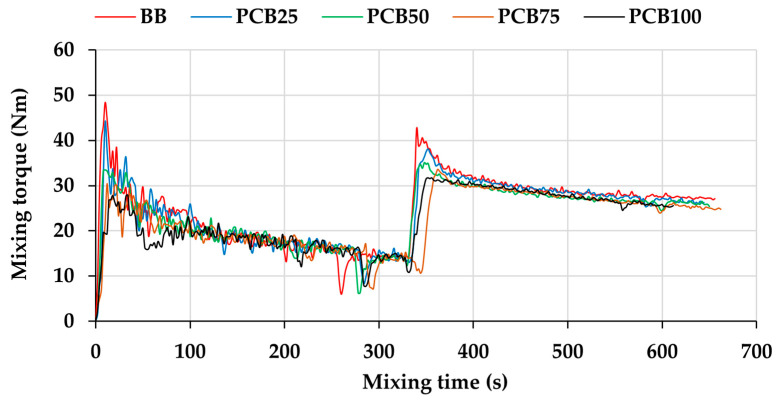
Curves of mixing torque.

The first parameter measured in the mixing process is the mixing torque (MT, Figure 2), which indicates the viscosity of the prepared compound as well as the effects of individual raw materials added to the compound (hardener, softener, etc.) [27]. The first raw material entering the mixing process is the matrix (SMR 10), mainly due to the need for its plasticization, i.e., reducing its viscosity and increasing its ability to absorb other raw materials into its volume. The matrix enters the mixing process with laboratory temperature and high viscosity, which is reflected in a sharp increase in MT after its dosing. Under the influence of temperature and blade rotation speed, the matrix reduces its viscosity, and the mixing torque begins to gradually decrease. A significant, albeit short-term, effect is recorded after the addition of stearic acid (SA) to the compound. Under the influence of temperature, SA changes its state from solid to liquid, which briefly reduces the viscosity of the compound, as well as reduces frictional forces directly in the mixing chamber [28]. However, by incorporating SA, its effect on MT disappears, and the mixing torque abruptly returns to a slightly lower value than before SA addition.

Comparing the individual torque curves of the prepared rubber compounds, significant differences are recorded precisely in the area corresponding to the addition of fillers. The basic compound achieved the highest value of the maximum MT due to the highest content of the commonly used filler CB N339, whose stiffening effect on the rubber compound is perfectly described [29]. The alternative filler EG in the BB compound does not have a significant stiffening effect, and its effect is more visible in other properties (e.g., tensile properties). With a gradual decrease in the CB content in the compound (in the interval from compound PCB_25_ to PCB_100_), the value of the maximum torque in the filler addition region decreases. This maximum expresses the initial stiffening effect of the compound and the nature of the fillers used. Pyrolytic carbon black (PCB), even in the case of using the smallest possible fraction (smaller than 25 μm), does not have such a stiffening effect compared to CB N339. In addition, the compounds also contain the filler EG, which, as mentioned above, does not have a significant effect on the stiffness of the compound even in the presence of the PCB filler. After the entire volume of fillers is added to the compound, the phase of dispersion and distribution of filler particles in the compound begins, which is reflected in a gradual decrease in the MT values. In this area, the BB compound shows the most significant decrease, since the CB filler added in the form of agglomerates is quickly broken down into individual nanoparticles by mixing and dispersed evenly in the volume of the compound [30]. It is the substitution of CB for the PCB filler and the presence of the EG filler that causes the decrease, since alternative fillers are added in particulate (micrometric) form, and thus the agglomerate breaking phase is eliminated and, conversely, the beginning of the filler dispersion phase is accelerated. The last added raw materials (Sulfur + TBBS) do not have a significant effect on the MT value. The vulcanization system (VS) in the prepared compounds does not have a significant volume that would be sufficient to change the compound MT. If during mixing after adding the vulcanization system, an increase in the MT value occurs, this may be due to excessive vs. dosage, or at increased temperature and long mixing interval, the onset of the vulcanization process (so-called premature vulcanization of the compound).

**Figure 3:**
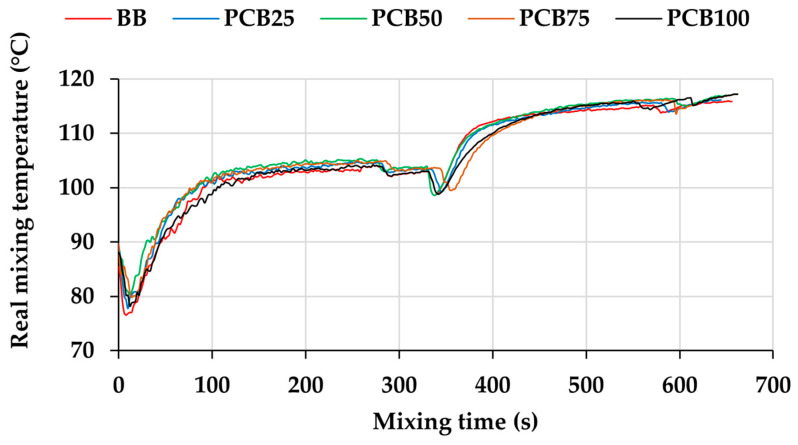
The real mixing temperature.

The second measurable parameter of the mixing process is the real mixing temperature (RMT), which forms the thermal history of the mixed compound (Figure 3). Due to the influence of the matrix entering the laboratory temperature into the mixing process, the RMT curve records a decrease, as the matrix receives heat from the surface of the mixing chamber, thereby cooling the chamber and at the same time cooling the sensor sensing the RMT of the mixed compound located directly below the dosing slot of the mixing device. Due to the influence of the chamber temperature and the blade rotation speed, the matrix quickly reaches the set mixing temperature value (in this case 90 °C) [31]. The mixing process reduces the viscosity of the matrix, which undergoes the process of plasticization, mainly by brakes of the rubber chains. Despite the decrease in viscosity and the associated decrease in resistance to the blade rotation or MT value (Figure 2), internal friction of the rubber chains occurs, which increases the RMT value [32]. The addition of stearic acid has a visible effect on the RMT curve, where a decrease is recorded. Stearic acid changes its state from solid to liquid under the influence of temperature, which reduces the friction of the matrix chains and overall friction in the mixing chamber [33].

By adding fillers (regardless of the ratio of N339 and PCB fillers), RMT begins to increase due to the increase in the viscosity of the compound and friction in the mixing chamber (also visible when recording the MT, Figure 1). By increasing the PCB content in the compound, a more gradual increase in RMT can be seen after PCB addition, which corresponds to a lower reinforcing effect of this filler. PCB, in combination with the second type of alternative filler, EG, forms a system of two fillers with large particles. As their content increases, the value of friction, which is caused by filler particles located between the chains of the matrix, decreases (in comparison with CB). RMT is especially important when adding the last raw materials, i.e., the vulcanization system, due to the risk of premature vulcanization (beginning of vulcanization) directly in the mixing chamber. Since the maximum RMT value decreases when the PCB content in the compound increases, the risk of premature vulcanization is reduced, which contributes to a safer and more stable mixing process. When replacing a commonly used filler with an alternative, RMT is important due to the relationship between compound viscosity and filler dispersion. While reducing the maximum RMT value when adding fillers is positive by minimizing the possibility of the onset of vulcanization, lower viscosity can cause agglomeration of fillers and their insufficient dispersion in the compound. The RMT value depends on the composition of the compound, but it is also possible to adjust it using the settings of the mixing process itself and thus achieve a curve with a similar course as in the case of the BB compound, which can have a positive effect on the properties of the compound [34,35].

### 3.2. Rheological Properties

**Figure 4:**
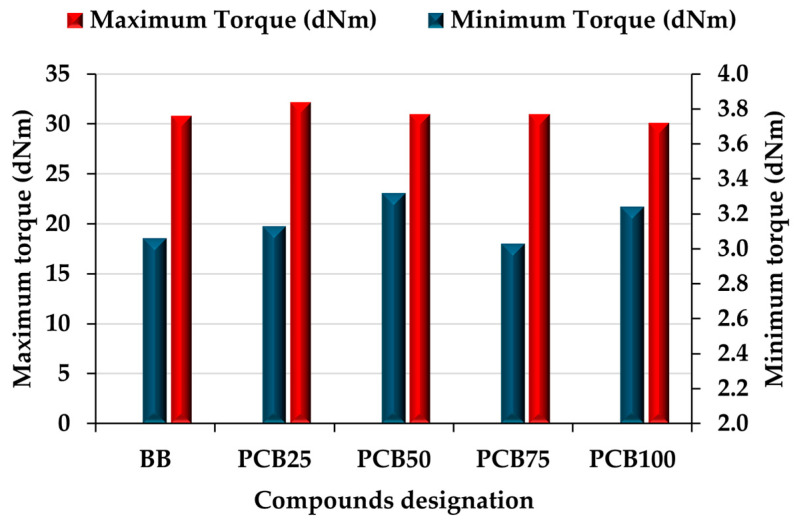
Rheological properties of prepared rubber compounds.

The rheological properties of rubber compounds (Figure 4) affect raw compounds operations such as calendering, cutting, and preparatory operations before the injection molding process from the point of view of economic costs (higher stiffness increases energy consumption for operations) as well as the quality of the resulting vulcanizate and operations associated with it (cutting samples, processing products). The minimum torque (M_L_), expressing the stiffness of the raw compound, showed differences with increasing PCB content at a maximum level of 9.6% [36]. The M_L_ results show that an increase was recorded for compounds PCB_25_ and PCB_50_. This effect, with respect to the parameters of the mixing process, can be attributed to a decrease in the mixing intensity (higher content of longer matrix chains) as well as to a still sufficient content of CB filler. The combination of the matrix chain length, the presence of large particles of EG and PCB fillers, and the highly stiffening CB filler will cause an increase in the M_L_ value. The presence of large particles of alternative fillers makes the motion of rubber chains more restricted (rubber chain without crosslinking), which can enhance the reinforcing effect of the CB filler [37,38]. A change in trend occurs in compound PCB_75_, whose M_L_ value has slightly decreased. This compound still contains part of the CB filler (25% of the original volume), and the alternative fillers EG and PCB begin to take over the majority effect.

The M_L_ value of compound PCB_75_ is almost identical to that of compound BB (reducing the CB filler content by 25% therefore resulting in a significant decrease). The reinforcement of the compound by the CB filler disappears, and since the PCB filler does not have such reinforcing properties, its content in the compound is not sufficient to maintain the trend of increasing M_L_. The last compound PCB_100_ contains exclusively alternative fillers and again records a slight increase in M_L_ with a value like that of compound PCB_50_ (difference of 2.5%). The lowest mixing intensity, in combination with a high content of alternative fillers and their particles, caused an increase in the M_L_ value, mainly by limiting the movement of rubber chains [39].

The maximum torque (M_H_), expressing the stiffness of the vulcanizate, as well as the efficiency of the vulcanization process itself, again shows minimal changes when replacing the CB N339 filler content with the alternative PCB filler (the most significant change in M_H_ values is at the level of max. 6.8%). The slight increase in the M_H_ value in the PCB_25_ compound can be attributed to the synergistic effect of the ratio of all CB, PCB, and EG fillers. The reduction in the content of the reinforcing CB filler, the low content of PCB filler in combination with the EG filler, facilitated the formation of a larger number of crosslinking bonds. By further increasing the PCB content at the expense of CB, the M_H_ value decreased slightly in the PCB_50_ (by 1.19 dNm) and PCB_75_ (by 1.17 dNm) compounds compared to the PCB_25_, due to the higher content of large particles of alternative fillers that limit the number of crosslinks. The effect is even more pronounced in the PCB_100_ compound. From the measured results of rheological properties, it can be concluded that the PCB filler does not have a significant impact on the values of rheological properties regardless of its content in the rubber compound, which is advantageous for the mentioned alternative filler PCB, since, from the point of view of rheological properties, it is a suitable substitute for the commonly used filler CB.

### 3.3. Vulcanization Characteristics

**Figure 5:**
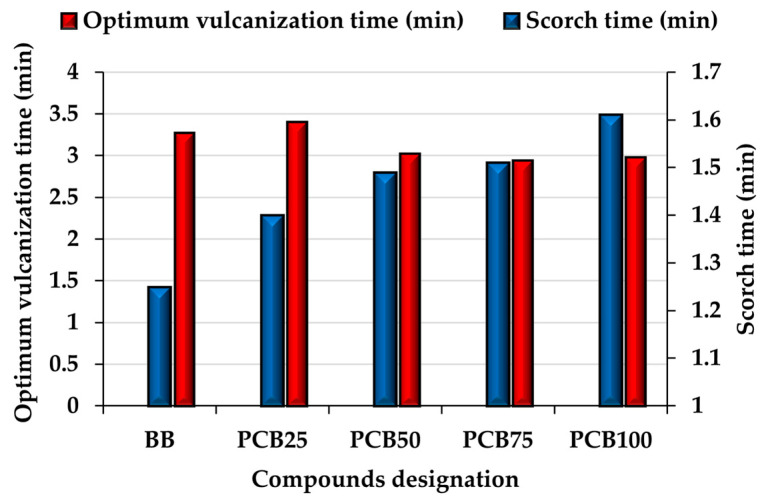
Vulcanization characteristic.

The most important properties of rubber compounds undoubtedly include their vulcanization characteristics (Figure 5), as they significantly affect not only the economic aspect in industrial applications, but also other properties investigated after the vulcanization process [40]. Scorch time (t_s02_), as the first of the vulcanization characteristics has a significant impact on the entire vulcanization process, as it indicates the value of the beginning of vulcanization and its value shortens/extends the initial induction part of the process, which can affect the total length of the vulcanization process [41,42]. Scorch time values increased with increasing PCB content in the rubber compound. This slowdown in the beginning of vulcanization can be attributed to several influences, whether alternative fillers or CB. Several publications mention the ability of the CB filler to support heat conduction from the surface of the compound to the volume, thereby supporting the activation of the vulcanization system. Gradual replacement of the CB volume with PCB in the presence of the EG filler slows down heat transfer and thus the activity of the vulcanization system (VS). Moreover, with the increasing content of PCB in the presence of the second type of alternative filler, EG, their micrometric particles form a real barrier for VS, which again contributes to the slowdown of the reaction itself. The most significant increase in the t_s02_ value compared to the basic compound BB is seen in compound PCB_25_ (25% replacement with PCB) and is 0.15 min. With a further increase in the content of PCB filler, the t_s02_ value increased more moderately. Compound PCB_100_, which contained exclusively alternative fillers, reached the highest value, since the absence of CB caused a slowdown in the overheating of the compound, and the high content of alternative filler particles also contributed to the slowdown of VS activation. The effect of PCB filler can therefore be changed by using a filler content with a lower maximum particle size, which also improves the preheating of rubber compounds and thus shortens the scorch time. The increase in t_s02_ can be considered positive, as the compounds have an extended time during which they can be processed at elevated temperature (including flow in the form of a vulcanizing press or injection molding). The effect of the PCB filler could be influenced by using its fraction with a lower maximum particle size, which would also improve the preheating of rubber compounds and thus shorten the scorch time [43].

Another vulcanization characteristic is the optimum vulcanization time (t_c90_), the value of which indicates the time required to achieve the desired crosslinking required for optimal mechanical properties and affects the length of the vulcanization process itself, thus directly affecting the economic aspect of production in industrial applications [44]. When 25% of the CB content was replaced by PCB (compound PCB_25_), the t_c90_ value increased (by 3.8%) compared to the BB compound, which can be attributed mainly to the effect of a denser vulcanization network as seen from the M_H_ results. If you were to calculate the time required to increase the M_H_ value by 1 dNm for the BB compound, the result would be 0.1062 min, and for compound PCB_25,_ again 0.1057 min, which shows that the increase in the t_c90_ value is only at the expense of the denser network for compound PCB_25_. With a further increase in the PCB filler content, the t_c90_ value decreases up to compound PCB_75_ (75% PCB content). This decrease is partly caused by a slight decrease in crosslinking density, but also by the PCB filler, which contains a higher percentage of sulfur (more than 3 times) compared to CB N339, which is used directly by the vulcanization system of the compound [45,46].

**Figure 6:**
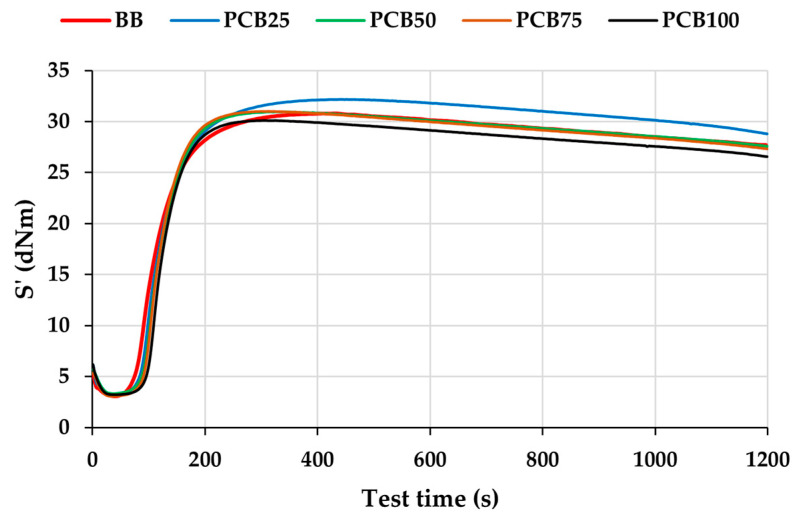
Isothermal vulcanization curves of prepared rubber compounds.

Isothermal vulcanization curves of the prepared compounds (Figure 6) exhibit a decrease in the induction phase, which is caused by the reduction in matrix viscosity due to exposure to the vulcanization temperature. At the minimum point (M_L_), all curves nearly overlap (maximum difference between curves is 0.32 dNm). With the onset of vulcanization, the curves rise sharply. The start of this rise, and thus the onset of the vulcanization process, depends on the scorch time, according to which the curves are arranged (in this area) in the order BB, PCB_25_, PCB_50_, PCB_75_, PCB_100_. An interesting observation is that with increasing PCB content in the compound, the steepness of the curves increases, which indicates an increased cure rate index (CRI). It was demonstrated that the alternative fillers PCB and EG can participate in the vulcanization process of rubber compounds and, through their sulfur content, promote an increase in CRI (Table 4). The highest maximum was achieved by the PCB_25_ compound. The curves of PCB_50_, BB, and PCB_75_ compounds showed the highest change in the maximum value at the level of 0.65%, resulting in mutual overlap. The PCB_100_ compound achieves the lowest maximum value. The post-cure region reveals a gradual decline of all compound curves, which is referred to as reversion. Studies report that the reason for reversion is the desulfurization of initially generated polysulfide crosslinks and the formation of more thermally stable di- and mono-sulfidic crosslinks [47,48].

If the conclusion given in the studies [47,48] is true, it is possible to calculate the ΔM value (by subtracting the torque value at the end of the test—M_End_ from the maximum torque value—M_H_) indicating the breakdown of polysulfide crosslinks and thus determine their relative representation in the compound as well as a possible increase or decrease due to the replacement of the CB filler (Table 4). The results show that the value expressing the relative representation of polysulfide crosslinks increases precisely with the gradual replacement of CB with PCB up to a content of 75%. It is therefore possible to assume that the replacement of the CB filler with an alternative filler PCB (in the presence of EG filler) affects the type of sulfur crosslink formed.

**Figure 7:**
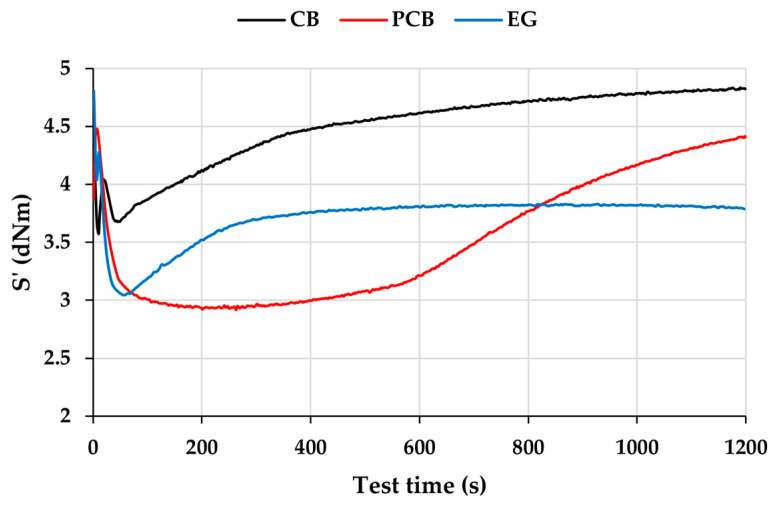
Isothermal vulcanization curves of rubber compounds without sulfur.

The vulcanization curves of compounds without vulcanizing agent content (Figure 7) show a significant difference in the behavior of individual fillers. For the conventional CB filler, a decrease is seen in the induction phase caused by heating the compound sample as well as by the disruption of the filler–filler network due to the oscillation of the rheometer disk. A more significant decrease is seen in the induction phase for alternative fillers PCB and EG, since these fillers do not form a filler–filler network like CB, and their particle size does not have such a stiffening effect; the decrease can be attributed to the viscosity of the matrix. After reaching the minimum S’, the CB and EG curves start to rise. The increase in the EG curve can be attributed to the sulfur content in the filler, since in industry it is formed during the flue gas desulfurization process, it can be assumed that free sulfur atoms are located on the surface of the particles and are therefore available for the crosslinking process [49]. In the CB compound, the increase is mainly caused by the filler–polymer reaction, like work [50]. An interesting finding is the development of the vulcanization curve for the PCB-filled compound, which, after a long induction phase, begins to slightly increase after exceeding 250 s and then increases rapidly after exceeding 600 s. The PCB filler contains sulfur, like the EG filler, which can enter the vulcanization process. The delayed change in the trend of the curve in the case of the PCB filler (compared to EG) may indicate a more difficult availability of sulfur for the vulcanization process [50].

### 3.4. Payne Effect

**Figure 8:**
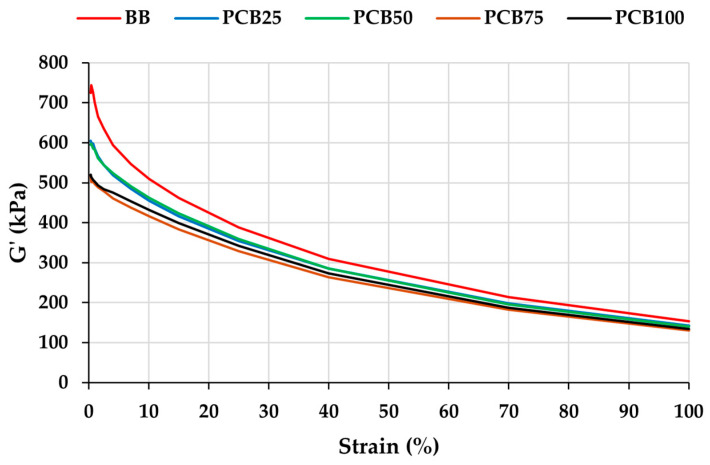
Strain-dependent G’ (Payne effect).

In the case of evaluating the Payne effect of rubber compounds (Figure 8), it is necessary to consider the type of filler (carbon black), which may be able to form its own filler–filler network in the compound [51]. The largest decrease in storage modulus (G′) is recorded for the BB compound. The decrease in the lower deformation amplitudes can also be attributed to the filler–filler interaction (formation of the filler network), which is disrupted by deformation [52]. By gradually replacing CB with PCB, a decrease in G′ values can be seen depending on the deformation amplitude. By reducing the CB content, their ability to form a filler–filler network is gradually limited, and thus the need for its disruption is also reduced. The curves indicate that, at contents between 50 and 75% in the compound, carbon black can affect the G′ values, whereas with contents below 50%, the amount is no longer sufficient to support the formation of a filler–filler network. In addition, by gradually increasing the PCB content, the dispersion effect of both types of alternative fillers increases, which is reflected in a decrease in G′ values [53,54]. The decrease in the Payne effect curves with increasing substitution of the conventional filler CB by PCB in the presence of EG indicates a good dispersion of filler. This improvement effect can also be attributed to the larger particle size of the alternative fillers PCB and EG (microparticle), since the particle number concentration per unit mass is lower compared to CB (nanoparticle).

Table 5 shows Payne effect values for the prepared compounds calculated from the difference between the moduli at 0.28% and 100% strain. From the difference in G’ values, it is possible to determine the degree of filler–filler interaction and therefore the possibility of filler agglomeration formation or improvement/deterioration of filler dispersion in the compound. A high value of ΔG’ indicates a higher filler–filler interaction and vice versa, a lower value indicates a low filler–filler interaction and improved filler dispersion. Gradual replacement of conventional CB filler by PCB, in the presence of EG filler, causes a decrease in ΔG’, which indicates a decreasing filler–filler interaction and good dispersion of fillers in the matrix (confirmed by morphology analysis) [55,56].

### 3.5. Hardness

**Figure 9:**
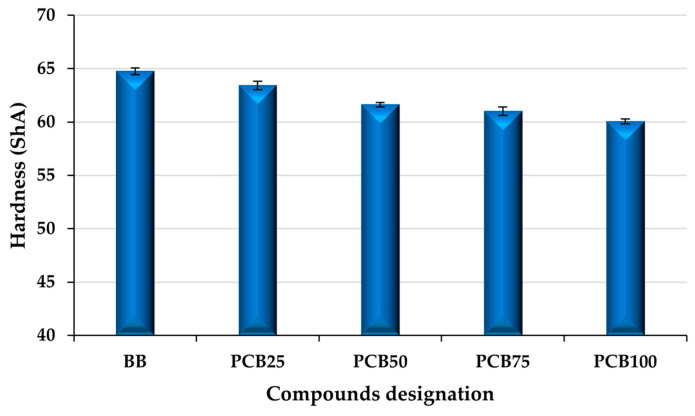
Hardness of prepared rubber compounds.

The hardness of rubber compounds (Figure 9) depends not only on the type of filler used (reinforcing, semi-reinforcing) but also on the crosslinking density created during vulcanization [57]. By replacing the CB filler content with the alternative PCB filler, the hardness value of the compounds decreases. Despite the high content of CB filler in relation to PCB in the presence of EG filler, the hardness value of compound PCB_25_ decreased by 1.32 ShA. This decrease was caused by the absence of the reinforcing CB filler content, as well as the above-mentioned increase in the content of flexible polysulfide crosslinks caused by the particle size of alternative fillers. It is the hardness value that confirms the claim about the formation of a larger number of polysulfide crosslinks in the presence of fillers with micrometric particles, which are more flexible and, despite higher crosslinking (see M_H_ value), show a lower hardness value [58,59].

A significant decrease (by 1.82 ShA) is seen in compound PCB_50_ compared to PCB_25_. The ratio of CB and PCB fillers is balanced (50:50), and a significant part of the impact on the decrease in hardness is taken over by the alternative fillers PCB and EG (lower reinforcing effect, larger particles). In addition, the crosslinking value of the compound has also decreased, and the impact of alternative fillers on the type of crosslinks formed is increasing. In other compounds (PCB_75_, PCB_100_), the trend of a decrease (more moderate) in hardness caused by the fillers PCB, EG, and their impact on the vulcanization process continues [60,61]. From the results, it can be concluded that the used PCB filler with a fraction smaller than 25 μm acts as a non-reinforcing filler. However, it should be noted again that the size of its particles has a significant influence, and in the case of using a fraction with a smaller maximum particle size, a significantly different effect can be achieved when using PCB. In the case of using a filler fraction in this study, the hardness value can be increased, for example, by adjusting the vulcanization system and increasing the crosslinking density of the sample, which may have negative consequences on the tensile properties [62].

### 3.6. Tensile Properties

**Figure 10:**
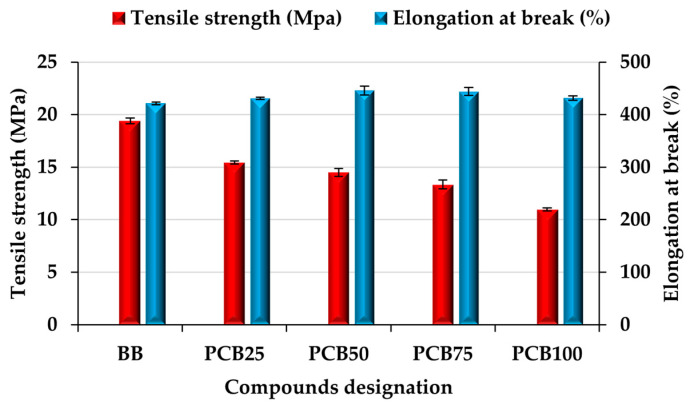
Tensile properties of prepared rubber compounds.

The tensile properties of the prepared rubber compounds (Figure 10) indicate the effect of replacing the CB N339 filler with an alternative filler, PCB, and the change in the resistance of the compound to tensile stress, as well as the interactions of the two types of alternative fillers, PCB and EG. An interesting finding is that the gradual replacement of the CB filler with a PCB filler does not have a significant effect on the elongation at break of the prepared compounds. The basic BB compound even reaches the lowest value among the prepared compounds due to the content of the CB filler, which, due to its particle size and the associated stiffening effect, limits the movement of the matrix chains linked by crosslinking bonds. It is possible to assume that the given value of the BB compound is, on the contrary, positively influenced by the alternative EG filler, which, due to its needle-like particle shape, partially limits the stiffening of the compound. Despite the proven entry of the EG filler into the vulcanization process, this filler is not cross-linked to such an extent with the matrix and gives space for the movement of its chains [63]. The PCB filler and its lower stiffening effect (compared to CB) and larger particle size, even in the presence of the second filler EG, also preserves the flexibility of the compound matrix (impact of changing the type of crosslinking). If the filler is well dispersed in the matrix, even with large particles, it preserves the chain mobility of the rubber matrix. However, maintaining flexibility is at the expense of the number of crosslinks, which are lower for large particles in the compound (see M_H_ values in Figure 4).

In addition, the content of micrometric particles in rubber compounds promotes the formation of a larger number of polysulfide crosslinks (C-Sx-C, where x = 3–6) mainly due to the reduction in the compound homogeneity. This phenomenon is a consequence of the less effective interaction of larger particles with rubber chains, which changes the chemical equilibrium during the formation of bonds. Therefore, in most cases of using alternative fillers, it is not a problem to achieve a high elongation at break value. The compounds containing the alternative fillers PCB and EG in this study, therefore, achieve a high elongation at break value, but at a low tensile strength value, since the flexible matrix and the absence of a reinforcing filler do not provide the matrix with strength. The mobility of the matrix is preserved; however, under stress and the influence of chain stretching during the test, a critical point is reached and the PCB and EG filler particles prevent further movement and cause them to rupture precisely in the area where the compounds should begin to show more significant resistance to tensile stress associated with an increase in the tensile strength value [64]. In addition, the higher content of polysulfide crosslinks, which are known for their flexibility and low strength compared to mono- and di-sulfide crosslinks, enhances this effect. With increasing content of alternative filler, the tensile strength value decreases, since there is a larger number of particles available that break the chains during ultimate chain stretching, as well as a higher number of polysulfide crosslinks in the vulcanizate. Again, the solution is to use a smaller fraction of the alternative filler PCB, which, in combination with the filler EG, would create a similar positive interaction as in the case of a BB compound containing CB and EG.

### 3.7. Morphology Analysis

**Figure 11:**
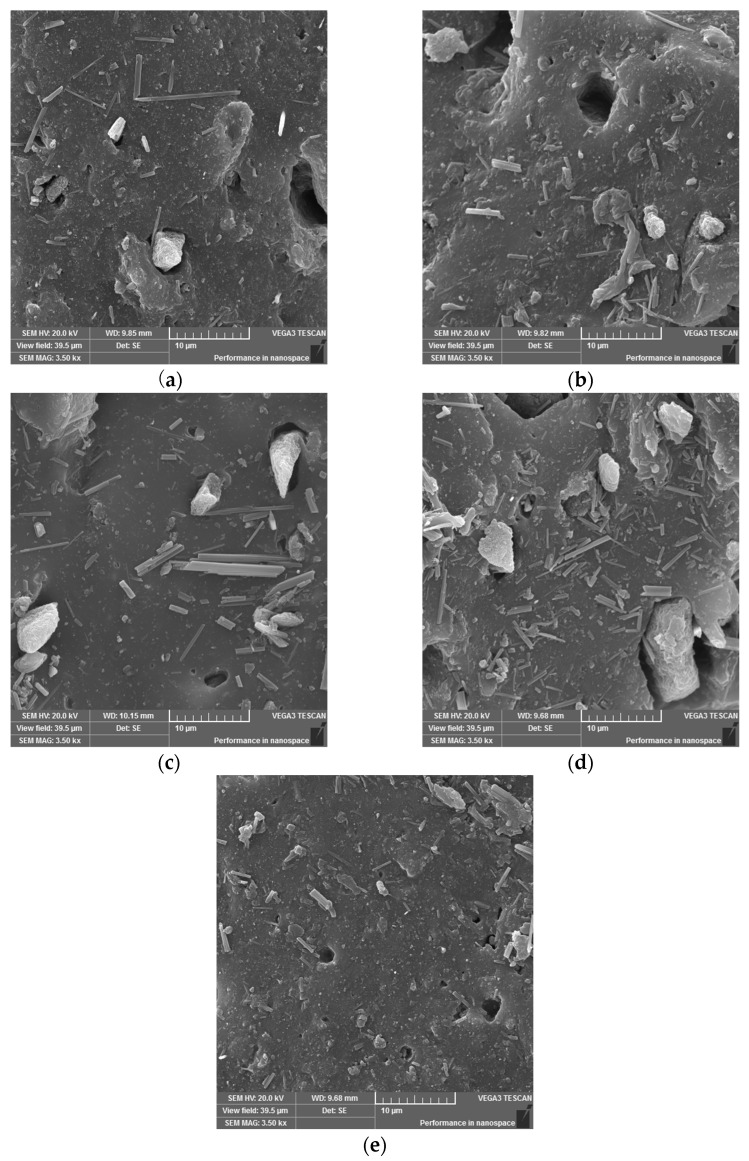
SEM scans of the cross-section surface of (**a**) PCB_25_; (**b**) PCB_50_; (**c**) PCB_75_; (**d**) PCB_100_; (**e**) BB (mag. 3500×).

Morphology analysis (Figure 11) confirmed the results of the Payne effect on good dispersion of fillers in the matrix with gradual replacement of CB content by alternative filler PCB. The filler particles do not show the formation of agglomerates, and there is no filler–filler interaction. Moreover, the scans of the compounds confirm the above-mentioned statement that alternative fillers are not crosslinked with the matrix, as the filler–matrix interface is clearly visible. The alternative fillers are therefore only mechanically mixed into the matrix, which may be caused by the size of their particles and the absence of a large specific surface area (and its activities) compared to conventional fillers. Improvement of the filler–matrix interaction can be achieved by using a smaller fraction of filler (especially PCB), which ensures an increase in the specific surface area, by modifying the filler, or by adding special additives to the compound (so-called coupling agents). By increasing the PCB content in the compound, an increasing occurrence of particles of this filler on the cross-section surface of the compound sample is recorded. In the case of the PCB_100_ compound, the occurrence of a larger number of smaller particles (needles) of the EG filler is also recorded, which can be justified by the presence of PCB particles, which have a larger particle size compared to CB and more intensively break the EG filler needles during mutual interactions in the mixing process. The black areas in the scans are voids formed due to particle loss during sample preparation, as these particles are not chemically cross-linked with the matrix.

## 4. Conclusions

The results of the study show that the alternative filler PCB affects the mixing process of rubber compounds, and with an increase in its content in the compound, the mixing intensity and the real mixing temperature of the compound decrease.

However, the parameters of the mixing process can be adjusted either by changing the set mixing parameters (rotation of the impellers, mixing temperature) or by changing the fraction of the alternative fillers used.

From the point of view of rheological properties, the gradual replacement of the CB content with the alternative filler PCB showed an effect mainly on the stiffness of the raw rubber compound, where an increase was recorded for compound PCB_50_ compared to the basic compound BB. The maximum torque, M_H,_ changed slightly with increasing PCB content compared to M_L_ relative to the basic compound BB. Changes in M_H_ were mainly caused by the alternative fillers PCB and EG, which, due to their content in the compound and particle size, changed the type of sulfur crosslinking bond formed.

The positive effect of the PCB filler in the presence of the second type of alternative filler, EG, was demonstrated on the scorch time values, where the t_s02_ value increased with increasing PCB content. The compounds, therefore, provided a longer time for processing at higher temperatures (160 °C) as well as time for filling the mold during the injection molding process. However, the increase in the t_s02_ value can also be changed by modifying the recipe (by incorporating a secondary vulcanization accelerator, the so-called Kicker). Despite the increase in t_s02_, the values of the optimal vulcanization time t_c90_ decreased for almost all compounds compared to the basic compound BB (except for compound PCB_25_, which showed higher crosslinking and therefore also a slightly higher time to achieve the desired degree of crosslinking). The shorter time required to achieve the optimal network density is positive, mainly from the point of view of the economic aspects of the industrial application of rubber compounds (a significant impact, especially in the vulcanization of products with a thick layer of the compound, where the t_c90_ time multiplies). Another reason the reduction in the t_c90_ value can be considered positive is that at the same time, the M_H_ value does not decrease, which would indicate a possible reduction in the crosslinking of the compound, and therefore it is possible to exclude the effect of reducing t_c90_ on the mechanical properties (especially tensile strength). The isothermal vulcanization curves of the compounds without the content of the vulcanizing agent confirmed the involvement of alternative fillers in the vulcanization process. The conclusion that alternative fillers affect the vulcanization process and increase the CRI was also confirmed by the isothermal vulcanization curves of the prepared compounds (in the range BB to PCB_100_). Using isothermal vulcanization curves and calculations from them, the influence of PCB and EG filler particles on the type of sulfur crosslinks was confirmed.

The Payne effect results showed a decrease in filler–filler interaction with increasing PCB content, in the presence of EG filler, in the compound. These results can be interpreted as maintaining good dispersion of fillers in the matrix.

The hardness of the studied compounds decreased with the gradual replacement of CB with PCB. Alternative fillers—along with their particle size—affected the hardness value, even when the density of the vulcanization network remained unchanged. With increasing PCB content in the presence of EG filler, the compounds become more flexible and therefore have lower resistance to the penetration of the hardness indenter.

From the point of view of mechanical properties, the replacement of the CB N339 filler with an alternative PCB filler in the presence of an EG filler maintains the flexibility of the matrix and achieves comparable (in the case of some compounds, higher) elongation at break values. On the contrary, the tensile strength decreases with increasing PCB content, since it has a larger particle size compared to the commonly used CB N339 filler, and in combination with the second type of alternative filler, EG, they constitute a significant limitation. As mentioned in the study, particles of alternative fillers often support the tearing of the compound matrix under tensile stress. The decrease in the tensile strength value can be prevented mainly by using a smaller particle size of the alternative PCB filler, since the second type of filler EG (despite the particle size) in combination with CB, did not cause a deterioration in the tensile strength, or simultaneously with the adjustment of the t_s02_ value by adjusting the recipe.

Summarizing the results of the study, it can be concluded that the PCB filler can be used as a substitute for the commonly used CB filler in a rubber compound containing two types of alternative fillers in terms of rheological properties, vulcanization characteristics, and elongation at break. Regarding the values of hardness and tensile strength, the application of rubber compounds containing a combination of two types of alternative fillers is still possible for products with lower mechanical loads, where high tensile strength or hardness is not required, and where it is sufficient to maintain flexibility and resistance under operating conditions, focusing on the ecological aspect and economic costs of production. The goal for the future is to use a fraction of PCB filler with a smaller particle size, as well as to study the process of particle size adjustment itself for the competitiveness of PCB filler compared to CB (operations to achieve smaller particle sizes constitute additional costs for the filler).

The study of the morphology of the cross-section surface of prepared compounds confirmed the conclusions obtained from the determination of the Payne effect about the preservation of good dispersion of fillers despite the increasing content of PCB filler at the expense of CB. Scans showed that PCB filler particles influence the reduction in the particle size of EG filler in the PCB100 compound, which also affects the dispersion of fillers in the matrix. The conclusion that alternative fillers are not crosslinked with the matrix was also confirmed, as loss of filler particles (occurrence of voids) was recorded due to sample preparation.

## Figures and Tables

**Table 1:** Recipes of prepared rubber compounds.

	Compounds Designation				
Ingredients	BB	PCB_25_	PCB_50_	PCB_75_	PCB_100_
	Content (phr)				
**SMR 10**	100	100	100	100	100
**CB N339**	**30.00**	**22.50**	**15.00**	**7.50**	**0.00**
**EG**	47	47	47	47	47
**PCB**	**0.00**	**7.50**	**15.00**	**22.50**	**30.00**
**Zinc oxide (ZnO)**	3	3	3	3	3
**Stearic acid (SA)**	2	2	2	2	2
**Sulfur Crystex OT33**	2.5	2.5	2.5	2.5	2.5
**TBBS**	1.5	1.5	1.5	1.5	1.5

**Table 2:** Chemical composition of used fillers.

Chemical Element	Filler Type		
	CB N399	EG	PCB
	Composition (wt.%)		
**C**	99.32	-	94.53
**Zn**	0.01	-	0.84
**S**	0.53	29.55	1.66
**Si**	0.10	1.16	2.31
**Ca**	0.02	68.64	0.66

**Table 3:** Recipes of prepared rubber compounds without sulfur.

	Compounds Designation		
Ingredients	CB	PCB	EG
	Content (phr)		
**SMR 10**	100	100	100
**CB N339**	**30**	**0**	**0**
**EG**	0	0	**47**
**PCB**	**0**	**30**	**0**
**Zinc oxide (ZnO)**	3	3	3
**Stearic acid (SA)**	2	2	2
**Sulfur Crystex OT33**	**0**	**0**	**0**
**TBBS**	1.5	1.5	1.5

**Table 4:** Properties calculated from isothermal vulcanization curves.

	Compounds Designation				
Properties	BB	PCB_25_	PCB_50_	PCB_75_	PCB_100_
**CRI (min^−1^)**	49.50	50.00	65.36	69.93	72.99
**Δ** **M (M_H_ − M_End_) (dNm)**	3.09	3.38	3.39	3.69	3.55

**Table 5:** Payne effect evaluation.

	Compounds Designation				
	BB	PCB_25_	PCB_50_	PCB_75_	PCB_100_
**G** **′ 0.28% (kPa)**	725.2	604.98	595.22	517.96	520.12
**G** **′ 100% (kPa)**	153.51	142.14	139.74	130.7	134.48
**ΔG** **′ (kPa)**	571.69	462.84	455.48	387.26	385.64

## Data Availability

The raw data supporting the conclusions of this article will be made available by the authors on request.
